# Effects of Extreme Temperatures on Cause-Specific Cardiovascular Mortality in China

**DOI:** 10.3390/ijerph121215042

**Published:** 2015-12-21

**Authors:** Xuying Wang, Guoxing Li, Liqun Liu, Dane Westerdahl, Xiaobin Jin, Xiaochuan Pan

**Affiliations:** 1 Department of Occupational and Environmental Health, School of Public Health, Peking University, Beijing 100191, China; wangxuying@bjmu.edu.cn (X.W.); liguoxing@bjmu.edu.cn (G.L.); liuliqun@bjmu.edu.cn (L.L.); jxb2000@sina.com (X.J.); 2 Sibley School of Mechanical and Aerospace Engineering, Cornell University, Ithaca, NY 14850, USA; danewest03@gmail.com

**Keywords:** extreme temperatures, cardiovascular disease, cerebrovascular disease, ischemic heart disease, hypertensive disease, distributed lag non-linear model, susceptibility

## Abstract

*Objective*: Limited evidence is available for the effects of extreme temperatures on cause-specific cardiovascular mortality in China. *Methods*: We collected data from Beijing and Shanghai, China, during 2007–2009, including the daily mortality of cardiovascular disease, cerebrovascular disease, ischemic heart disease and hypertensive disease, as well as air pollution concentrations and weather conditions. We used Poisson regression with a distributed lag non-linear model to examine the effects of extremely high and low ambient temperatures on cause-specific cardiovascular mortality. *Results*: For all cause-specific cardiovascular mortality, Beijing had stronger cold and hot effects than those in Shanghai. The cold effects on cause-specific cardiovascular mortality reached the strongest at lag 0–27, while the hot effects reached the strongest at lag 0–14. The effects of extremely low and high temperatures differed by mortality types in the two cities. Hypertensive disease in Beijing was particularly susceptible to both extremely high and low temperatures; while for Shanghai, people with ischemic heart disease showed the greatest relative risk (RRs = 1.16, 95% CI: 1.03, 1.34) to extremely low temperature. *Conclusion*: People with hypertensive disease were particularly susceptible to extremely low and high temperatures in Beijing. People with ischemic heart disease in Shanghai showed greater susceptibility to extremely cold days.

## 1. Introduction

Climate change is likely to cause an increasing trend in extreme temperatures worldwide [1]. Previous epidemiological studies have suggested that extreme temperatures are associated with peaks in morbidity and mortality [2,3,4]. The adverse effects of extreme temperatures are strongly apparent in people whose health is already compromised [5]. Cardiovascular diseases, especially ischemic heart disease and cerebrovascular disease, have remained as the top major killers during the past decade in many countries and account for a large proportion of the total burden of diseases [6].

Generally, the relationship between temperature and mortality was U, J or V shaped with increased mortality at both high and low temperatures [7,8]. It has been demonstrated that the adverse effects of extremely high and low temperatures may vary widely depending on the vulnerability of the population and climatic types [7,9,10,11]. Many studies have examined the association between ambient temperature and cardiovascular disease. Some of them investigated the effects of temperature on single cardiovascular mortality, such as stroke or ischemic heart disease in multiple cities and then combined the effects by using meta-analysis [12,13,14,15]. Others focused on a single city and examined the effects of temperature on the deaths from cardiorespiratory disease [16,17]. However, medical conditions may confer susceptibility to extreme temperatures [18,19]. Furthermore, the association between cause-specific cardiovascular mortality and ambient temperature in different areas still remains unclear. Only a few studies have examined the susceptibility of cause-specific cardiovascular mortality related to extreme temperatures [5,19,20,21]. Moreover, their results are inconsistent [5,21]. In addition, even in the same country, the impact of extreme temperatures differed by regional climatic types [22]. Hence, there is a need to explore the associations between extreme temperatures and cause-specific cardiovascular mortality in two Chinese cities with different climates. Such a study could provide important evidence for identifying susceptible people with specific diseases.

In this study, we aimed to examine the associations between extreme temperatures and the population mortality of cardiovascular disease (CVD), cerebrovascular disease (CBD), ischemic heart disease (IHD) and hypertensive disease (HPD) in Beijing and Shanghai, China. The findings of this study will improve our understanding of the susceptibility characteristics of different regions and cause-specific cardiovascular diseases related to extreme temperatures.

## 2. Materials and Methods

### 2.1. Study Areas

Beijing, located in northern China with a latitude of 39″26′–41″03′ N, is the capital and second largest Chinese city. In 2009, there were over 17 million permanent residents in Beijing. It has a rather dry, monsoon-influenced continental climate and four distinct seasons with cold, windy and dry winters and hot, rainy summers. Shanghai, located in southeastern China with a latitude of 30″40′–31″53′ N, is the largest city by population in China, with over 19 million permanent residents in 2009. It has a humid subtropical climate, which is characterized by short, cool winters with mild days and long, hot summers.

The main reasons why we chose these two cities were as follows: First, they have different climate characteristics. Beijing has a temperate continental climate, while Shanghai has a subtropical monsoon climate. Second, while the climate differs, there are several similarities between the two cities. They are metropolitan cities, and their feature characteristics, such as age structure, education level and life expectancy, are comparable (Table A1).

### 2.2. Mortality Data

Daily primary causes of death data from 1 January 2007–31 December 2009 were obtained from the Center for Public Health Surveillance and Information Service of China Centre for Disease Control and Prevention (China CDC). In each city, the health service centers and hospitals filled out the death certificates for each person on the primary causes. Then, trained workers checked and confirmed the causes of death for each person and reported to the China CDC through the network. Beginning in 2004, the Chinese CDC electronically archived all death certificates all around the country. The data were validated each year by China CDC. Therefore, the daily primary causes of death data in each city could represent the population mortality of each city. Additionally, the mortality data included the information of the death date, address, age, gender and cause of death. Causes of deaths were classified according to the International Classification of Diseases, 10th Revision (ICD-10). The corresponding ICD-10 codes for CVD, CBD, IHD and HPD are I00-I69, I60-I69, I20-I25 and I10-I15, respectively. This study was approved by the Ethics Committee of Peking University Health Science Centre, Beijing, China.

### 2.3. Meteorological and Air Pollution Data

Daily meteorological data during 2007–2009 were obtained from the China Meteorological Data Sharing Service System for the two cities, including relative humidity (RH), wind speed, maximum temperature, minimum temperature and mean temperature (MT). The meteorological stations are located in the urban district of each city. Daily ambient concentrations of air quality data for the same period were obtained from the local Environmental Monitoring Center. These pollution monitoring stations were distributed throughout each city (12 in Beijing and 17 in Shanghai). The city-wide daily average concentrations of particle matter of a median aerodynamic diameter less than 10 microns (PM_10_), sulfur dioxide (SO_2_) and nitrogen dioxide (NO_2_) were averaged from the daily mean concentration at monitoring stations in each city.

### 2.4. Statistical Analysis

We applied a time series method combined with a Poisson regression model to examine the relationship between temperature and mortality. We assumed that the association between temperature and cause-specific cardiovascular mortality was non-linear [16]. Previous studies [23,24] indicated the delayed effects of temperature on mortality. Therefore, a distributed lag non-linear model (DLNM) incorporated in a Poisson regression was used to explore the non-linear and delayed effects of temperature on daily death counts.

(1) $$Log\left[E\left(Yt\right)\right]=\alpha +\beta Tt,l+ns\left(time,4/year\right)+ns\left(RH_{t},3\right)+ns\left(Wind_{t},\;3\right)+ns\left(PM_{10t},3\right)\;+ns\left(NO_{2t},3\right)+ns\left(SO_{2t},3\right)+\;\eta DOW_{t}\;$$

where *t* is the date of the observation; *Y* is the observed daily death counts on day *t*; *α* is the intercept; *ns* (...) is a natural cubic spline. A natural cubic spline with four degrees of freedom for time was used to control the time trend and seasonality [25]. RH_t_, Wind_t_, PM_10t_, SO_2t_ and NO_2t_ represent the relative humidity, wind speed and concentrations of PM_10_, SO_2_ and NO_2_ on day t, and the degrees of their freedom are 3. DOW_t_ is the day of the week on day *t*, and *η* is the vector of coefficients. MT_t,1_ is the matrix obtained by applying the DLNM to mean temperature. β is the vector of coefficients for MT_t,1_. *l* is the lag day for mean temperature. A maximum lag of 27 days was used to examine the delayed effect of temperature on cause-specific cardiovascular mortality [26,27]. The degrees of freedom for mean temperature and lag were then selected by the minimum value of the Akaike information criterion (AIC) [28] for the Poisson model. In the final model, we used natural cubic splines with five degrees of freedom for temperature and four degrees of freedom for lag. The reference temperature for the three-dimensional plot was the mean temperature of each city.

We defined the 1st and 99th percentile of the temperature of two cities as the extremely low and extremely high temperatures, respectively. To quantify the effects of extreme temperatures, we calculated the relative risks for cause-specific cardiovascular mortality at the 1st percentile of temperature compared to the 10th percentile of temperature as the cold effect and the 99th percentile of temperature compared to the 90th percentile of temperature as the hot effect, respectively. In order to estimate the cumulative effects of extreme temperatures, we calculated the lag effects along lags of 0, 0–7, 0–14 and 0–27. To examine the seasonal effects of the extreme temperatures, we stratified the season into cold period (November–March) and warm period (April–October). The central heating system in Beijing works every year from November–March. However, Shanghai is one of the cities without a central heating system during this period. We also analyzed the effects of both extremely low and high temperature for those aged ≥65 years old.

**Figure A1:**
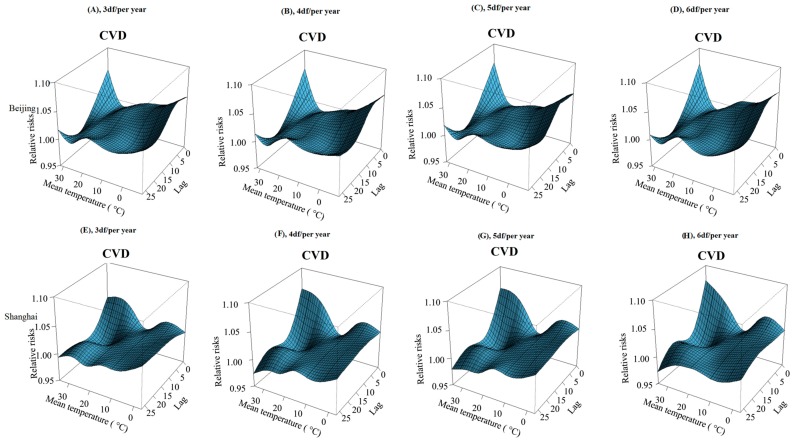
Three-dimensional graph of the relative risks for mortality from cardiovascular disease by temperature (°C) and lag days during 2007 and 2009 in Beijing and Shanghai, China. (**A**–**D**) Three to 6 degrees of freedom (df) per year for the time trend in Beijing, respectively; (**E**–**H**) Three to six df per year for the time trend in Shanghai, respectively.

**Figure A2:**
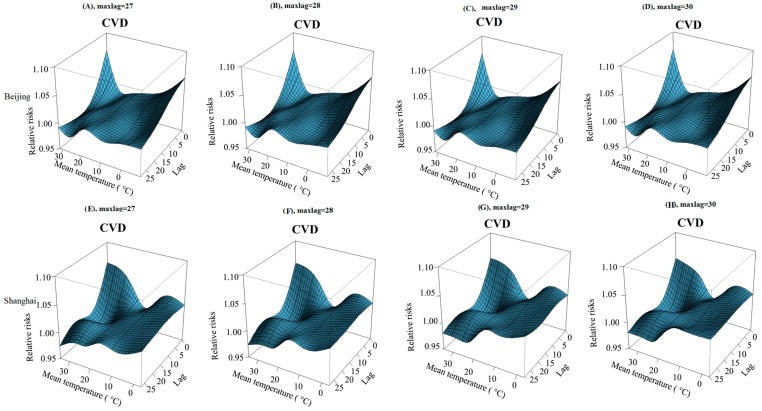
Three-dimensional graph of the relative risks for mortality from cardiovascular disease by temperature (°C) and lag days during 2007 and 2009 in Beijing and Shanghai, China. (**A**–**D**) A maximum lag of 27–30 days for mean temperature in Beijing, respectively; (**E**–**H**) A maximum lag of 27–30 days for mean temperature in Shanghai, respectively.

**Figure A3:**
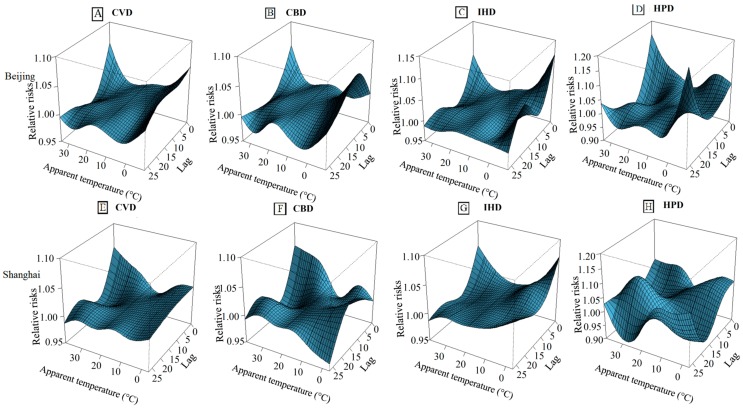
Three-dimensional graph of the relative risks for cause-specific cardiovascular disease by apparent temperature (°C) and lag days during 2007 and 2009 in Beijing and Shanghai, China. (**A**–**D**) The relative risks for CVD, CBD, IHD and HPD mortality in Beijing, respectively; (**E**–**H**) The relative risks for mortality from CVD, CBD, IHD and HPD mortality in Shanghai, respectively.

**Figure A4:**
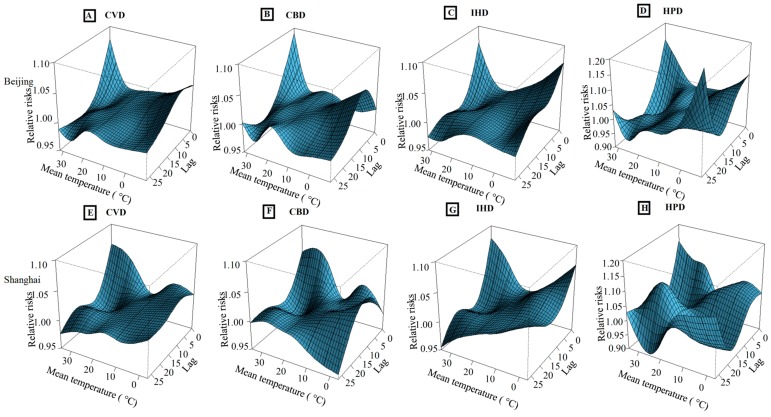
Three-dimensional graph of the relative risks for cause specific cardiovascular disease by mean temperature (°C) and lag days during 2007 and 2009 in Beijing and Shanghai, China (excluding the air pollution in the model)*. Note: * the effects of temperature on mortality from cardiovascular and cerebrovascular disease in two cities were excluded the influences of air pollution. The reference temperatures were 13.6 °C for Beijing and 17.7 °C for Shanghai, respectively; (**A**–**D**) represent the relative risks for mortality from CVD, CBD, IHD and HPD in Beijing, respectively; (**E**–**H**) represent the relative risks for mortality from CVD, CBD, IHD and HPD in Shanghai, respectively.

**Figure A5:**
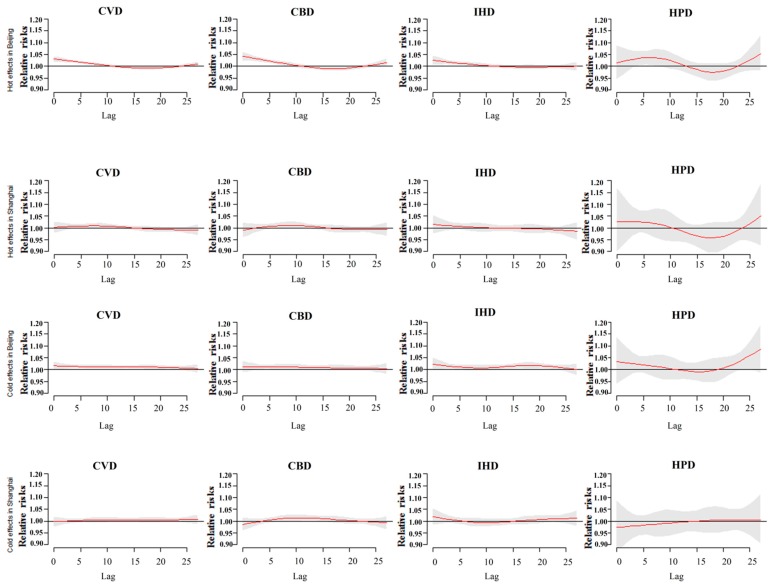
The hot and cold effects on cause-specific cardiovascular mortality along lag days.

Sensitivity analyses were conducted by changing the degrees of freedom (3–6 per year) for time and lag days (Figure A1 and Figure A2), using the CVD mortality as the example. We also refitted models by using the 25th percentile as the cold threshold and the 75th percentile as the hot threshold to calculate cold and hot effects, respectively (Table A2 and Table A3). In addition, we also performed the effects of apparent temperature on mortality (Figure A3). The sensitivity analysis of excluding the air pollution in the model was also performed (Figure A4), as well as the cold and hot effects on cause-specific cardiovascular mortality along lag days (Figure A5). The R software (Version 3.1.2) was used to fit all models. The DLNM package [21,22] in R was used to simultaneously smooth temperature and lags using a natural cubic spline.

## 3. Results

### 3.1. Descriptive Statistics

The daily cause-specific cardiovascular mortality during 2007–2009 in Beijing and Shanghai is shown in Table 1. The daily death counts for CBD were 40 and 18 in Beijing and Shanghai, respectively, which took the biggest proportion in all cause-specific cardiovascular diseases. IHD followed and HPD occupied the smallest percentage. Beijing had greater numbers of deaths for cause-specific cardiovascular diseases than Shanghai. A higher mean temperature (17.7 °C *vs*. 13.6 °C) and a higher relative humidity (69.8% *vs*. 52.5%) were observed in Shanghai (Table 2). In addition, Beijing had higher concentrations of PM_10_ and NO_2_ than Shanghai.

**Table 1:** Distribution of daily cause-specific cardiovascular mortality in Beijing and Shanghai, China, during 2007–2009. CVD, cardiovascular disease; CBD, cerebrovascular disease; IHD, ischemic heart disease; HPD, hypertensive disease.

City	Cause-Specific Cardiovascular	Group	Mean ± SD	Minimum	25%	Median	75%	Maximum
Beijing	CVD	All ages	87 ± 18	49	73	84	100	154
		Aged ≥ 65	70 ± 16	34	58	68	81	127
	CBD	All ages	40 ± 9	18	33	39	46	77
		Aged ≥ 65	32 ± 8	12	27	32	38	64
	IHD	All ages	38 ± 9	13	31	37	45	75
		Aged ≥ 65	31 ± 9	10	25	31	37	67
	HPD	All ages	2 ± 1	0	1	2	3	12
		Aged ≥ 65	2 ± 1	0	1	2	3	9
Shanghai	CVD	All ages	35 ± 8	14	29	34	40	65
		Aged ≥ 65	30 ± 8	11	24	29	35	61
	CBD	All ages	18 ± 5	6	14	17	21	34
		Aged ≥ 65	15 ± 4	4	12	14	18	30
	IHD	All ages	13 ± 4	2	9	12	15	28
		Aged ≥ 65	11 ± 4	1	9	11	14	27
	HPD	All ages	2 ± 1	0	1	1	2	6
		Aged ≥ 65	1 ± 1	0	0	1	2	6

**Table 2:** Summary statistics of weather conditions and air pollutants during 2007–2009 in Beijing and Shanghai, China.

City	Variables	Mean ± SD	Min			Percentiles					Max
				1th	10th	25th	50th	75th	90th	99th	
Beijing	Mean temperature (°C)	13.6 ± 10.9	−9.4	−6.1	−1.5	3.0	15.1	24.1	26.9	29.8	31.4
	Relative humidity (%)	52.5 ± 20.3	11.0	16.0	24.0	36.0	53.0	69.0	79.0	90.0	97.0
	PM_10_ (µg/m^3^)	131.2 ± 8.2	7.0	17.9	45.5	74.0	116.0	162.0	238.0	403.3	600.0
	SO_2_ (µg/m^3^)	39.9 ± 4.1	6.0	6.0	9.0	12.0	23.0	52.0	98.0	189.0	248.0
	NO_2_ (µg/m^3^)	57.1 ± 2.4	10.0	19.2	28.8	40.0	52.8	68.8	88.8	132.8	152.0
Shanghai	Mean temperature (°C)	17.7 ± 8.9	−3.4	0.5	5.3	9.8	18.7	25.2	28.9	32.4	34.6
	Relative humidity (%)	69.8 ± 12.2	30.0	38.0	53.0	62.0	71.0	79.0	84.5	91.0	95.0
	PM_10_ (µg/m^3^)	84.4 ± 5.3	12.0	21.9	34.0	48.0	74.0	106.0	147.0	236.4	600.0
	SO_2_ (µg/m^3^)	47.4 ± 2.9	10.0	12.0	18.0	26.0	40.0	62.0	86.0	138.2	235.0
	NO_2_ (µg/m^3^)	55.0 ± 2.1	13.0	16.0	30.4	40.0	53.0	67.0	85.0	115.2	146

### 3.2. The Non-Linear Relationship between Mean Temperature and Cause-Specific Cardiovascular Mortality

**Figure 1:**
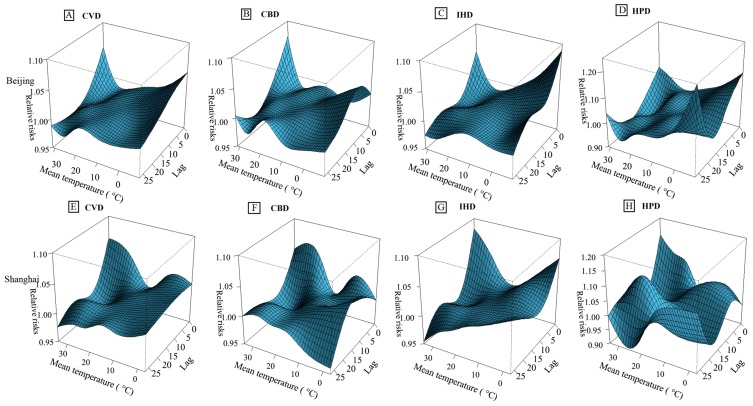
Three-dimensional graph of the relative risks for cause-specific cardiovascular mortality by temperature (°C) and lag days in Beijing and Shanghai, China, during 2007–2009. (**A**–**D**) The relative risks for mortality of CVD, CBD, IHD and HPD in Beijing, respectively; (**E**–**H**) The relative risks for mortality of CVD, CBD, IHD and HPD in Shanghai, respectively. The relative risks used five degrees of freedom for temperature and four degrees of freedom for lag up to 27 days. The reference temperatures were 13.6 °C for Beijing and 17.7 °C for Shanghai, respectively.

The three-dimensional exposure-response surfaces of mean temperature on cause-specific cardiovascular mortality along lag days are shown in Figure 1. In general, the estimated effects of temperatures on cause-specific cardiovascular mortality were nonlinear in the two cities, with increased mortality rates on days with both the high and low temperatures. For CVD mortality, both the cold and hot effects in the two cities reached the largest at lag 0 and decreased along with the lag days. For CBD morality, the cold effects in Beijing increased from lag 0–lag 5 day, with a peak on lag 5, whereas the cold effects in Shanghai increased from lag 0–lag 10 days, with the peak on lag 10. For IHD mortality, both the cold and hot effects in Beijing decreased along with the lag days; while the cold effects in Shanghai decreased from lag 0–lag 10 and then increased markedly after lag 10. For HPD mortality, both the cold and hot effects in the two cities increased after lag 20. In general, the hot effects appeared immediately and persisted at least 10 days, while cold effects last longer. The cold effects on HPD mortality in Beijing and IHD mortality in Shanghai lasted longer than the cold effects on other cardiovascular diseases.

**Figure 2:**
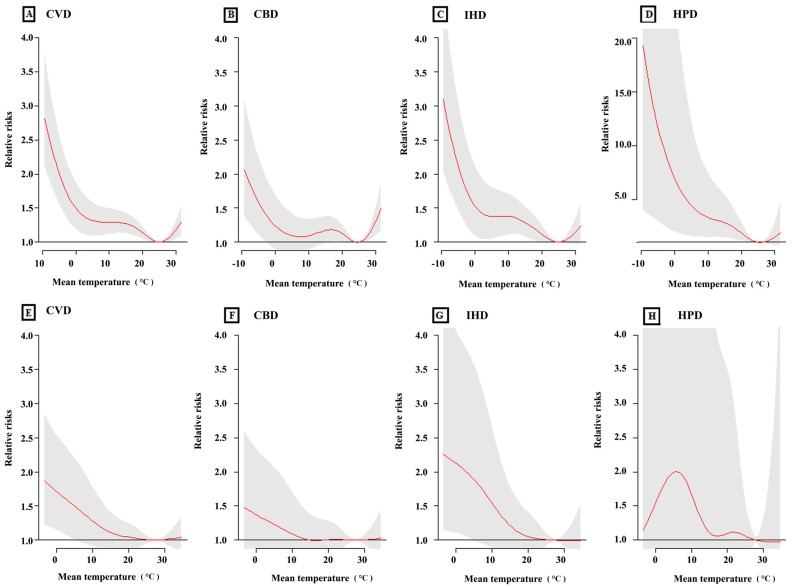
The relationship between mean temperature and cause-specific cardiovascular mortality over lag 0–27 days in Beijing and Shanghai, China, during 2007–2009. (**A**–**D**) The relationship between mean temperature and CVD, CBD, IHD and HPD in Beijing, respectively; (**E**–**H**) The relationship between mean temperature and CVD, CBD, IHD and HPD in Shanghai, respectively. The maximum likelihood estimate of relative risks is shown as smooth red lines, and the point-wise 95% confidence intervals are shown in the gray regions.

Figure 2 shows the cumulative relative risks of cause-specific cardiovascular mortality associated with mean temperature over the 27 lag days. It demonstrated that both the high and low temperatures increased the relative risks for cause-specific cardiovascular mortality in Beijing, but only the low temperatures increased cause-specific cardiovascular mortality in Shanghai. We should note that HPD mortality in Shanghai seemed not to be sensitive to low temperatures. In general, low temperature was associated with greater risk of cause-specific cardiovascular mortality in comparison with that of high temperature. The optimum temperatures for mortalities in Beijing and Shanghai were 25 °C and 28 °C, respectively.

### 3.3. The Cumulative Hot and Cold Effects of Temperature on Cause-Specific Cardiovascular Mortality

The cumulative hot effects on cause-specific cardiovascular mortality associated with the 99th percentile of temperature against the 90th percentile of temperature at lag 0, lag 0–7, lag 0–14 and lag 0–27 days in the two cities are shown in Table 3. The hot effects on cause-specific cardiovascular mortality reached the largest at lag 0–14 days in the two cities. The cumulative hot effects on cause-specific cardiovascular mortality in Beijing at lag 0–14 were statistically significant (*p* < 0.05). However, for Shanghai, the cumulative hot effects were not statistically significant (*p* > 0.05). From Table 3, we also can find that the cumulative hot effects on cause-specific cardiovascular mortality in Beijing were higher than those in Shanghai. Additionally, for HPD mortality, the cumulative effects at lag 0–14 in Beijing were the strongest (relative risks (RRs) = 1.39, 95% CI: 1.01, 1.92).

**Table 3:** The cumulative hot effects (relative risks (RRs) and 95% CI) on mortality from cardiovascular disease, cerebrovascular disease, ischemic heart disease and hypertensive disease of Beijing and Shanghai, China.

City	Lag	Hot Effects * (Relative Risks and 95% Confidence Intervals)			
		CVD	CBD	IHD	HPD
Beijing	0	1.02 (1.01, 1.04) ^a^	1.04 (1.02, 1.06) ^a^	1.02 (1.00, 1.04) ^a^	1.01 (0.94, 1.08)
	0–7	1.17 (1.12, 1.22) ^a^	1.24 (1.16, 1.32) ^a^	1.13 (1.06, 1.21) ^a^	1.25 (0.96, 1.61)
	0–14	1.18 (1.11, 1.25) ^a^	1.25 (1.15, 1.34) ^a^	1.15 (1.05, 1.24) ^a^	1.39 (1.01, 1.92) ^a^
	0–27	1.13 (1.04, 1.22) ^a^	1.21 (1.08, 1.35) ^a^	1.11 (0.98, 1.24)	1.36 (0.87, 2.14)
Shanghai	0	1.00 (0.98, 1.02)	1.00 (0.96, 1.02)	1.01 (0.97, 1.05)	1.01 (0.90, 1.16)
	0–7	1.05 (0.97, 1.14)	1.01 (0.91, 1.12)	1.07 (0.94, 1.22)	1.21 (0.79, 1.86)
	0–14	1.10 (0.99, 1.20)	1.07 (0.95, 1.21)	1.08 (0.93, 1.25)	1.21 (0.71, 1.93)
	0–27	1.02 (0.89, 1.16)	1.01 (0.85, 1.20)	0.99 (0.80, 1.23)	0.97 (0.47, 2.01)

Notes: * The relative risks of cause-specific cardiovascular mortality were associated with the 99th percentile of temperature against the 90th percentile of temperature. The 99th percentile of temperature in Beijing and Shanghai was 29.8 °C and 32.4 °C, respectively. The 90th percentile of temperature in Beijing and Shanghai was 26.9 °C and 28.9 °C, respectively; ^a^ *p* < 0.05.

Table 4 shows the cumulative cold effects on cause-specific cardiovascular mortality at lag 0, lag 0–7, lag 0–14 and lag 0–27 days in the two cities. The cumulative cold effects on cause-specific cardiovascular mortality reached the largest at lag 0–27 days. The cumulative cold effects on HPD mortality at lag 0–27 in Beijing were the strongest (RRs = 1.64, 95% CI: 1.06, 2.55) for the cause-specific cardiovascular mortality; while for Shanghai, people with ischemic heart disease showed higher susceptibility to the extremely low temperatures than the other types of cardiovascular disease. Both the hot and cold effects on cause-specific cardiovascular mortality in Beijing along lag days were stronger than those in Shanghai. At lag 0–27, the cold effects were greater than the hot effects for all cause-specific cardiovascular diseases in the two cities.

### 3.4. The Stratification of Seasonal Analysis for the Cumulative Hot and Cold Effects on Mortality from Cause-Specific Cardiovascular Diseases

The seasonal analysis for the cumulative hot and cold effects on cause-specific cardiovascular morality is shown in Table 5. The cold effects in Beijing became weaker during the cold period compared to the whole-year analysis. However, stronger hot effects during the warm period in Beijing were observed. For Shanghai, the seasonal analysis results were comparable with the whole-year analysis. Both the hot and cold effects in Beijing were still slightly higher than those in Shanghai.

**Table 4:** The cumulative cold effects on cardiovascular disease, cerebrovascular disease, ischemic heart disease and hypertensive disease of Beijing and Shanghai, China.

City	Lag	Cold Effects * (Relative Risks and 95% Confidence Intervals)			
		CVD	CBD	IHD	HPD
Beijing	0	1.01 (0.99, 1.03)	1.01 (0.98, 1.03)	1.02 (0.99, 1.04)	1.03 (0.93, 1.13)
	0–7	1.11 (1.05, 1.17) ^a^	1.10 (1.02, 1.19) ^a^	1.12 (1.03, 1.21) ^a^	1.21 (0.88, 1.65)
	0–14	1.21 (1.13, 1.28) ^a^	1.18 (1.07, 1.29) ^a^	1.19 (1.09, 1.31) ^a^	1.22 (0.85, 1.74)
	0–27	1.35 (1.24, 1.46) ^a^	1.27 (1.14, 1.42) ^a^	1.39 (1.24, 1.57) ^a^	1.64 (1.06, 2.55) ^a^
Shanghai	0	0.99 (0.97, 1.02)	0.98 (0.96, 1.01)	1.01 (0.98, 1.05)	0.97 (0.87, 1.08)
	0–7	1.01 (0.94, 1.09)	0.99 (0.90, 1.09)	1.05 (0.94, 1.18)	0.85 (0.57, 1.25)
	0–14	1.05 (0.97, 1.14)	1.08 (0.97, 1.21)	1.02 (0.89, 1.16)	0.81 (0.52, 1.27)
	0–27	1.14 (1.02, 1.27) ^a^	1.11 (0.95, 1.29)	1.16 (1.03, 1.34) ^a^	0.86 (0.47, 1.57)

Notes: * The relative risks of cause-specific cardiovascular mortality were associated with the 1st percentile of temperature against the 10th percentile of temperature. The 1st percentile of temperature in Beijing and Shanghai was −6.1 °C and 0.5 °C, respectively. The 10th percentile of temperature in Beijing and Shanghai was −1.5 °C and 5.3 °C, respectively; ^a^ *p* < 0.05.

**Table 5:** The cumulative hot effects during the warm period at lag 0–14 and cold effects during the cold period at lag 0–27 days in Beijing and Shanghai, China.

City	Cause	Hot Effects Lag 0–14 (Warm Period)	Cold Effects Lag 0–27 (Cold Period)
Beijing	CVD	1.32 (1.16,1.51) ^a^	1.35 (0.80,2.26)
	CBD	1.31 (1.09,1.57) ^a^	1.11 (0.52,2.36)
	IHD	1.33 (1.08,1.62) ^a^	1.36 (0.74,3.38)
	HPD	3.04 (1.42,6.48) ^a^	1.56 (0.02,8.70)
Shanghai	CVD	1.14 (0.93,1.40)	1.07 (0.60,1.90)
	CBD	1.14 (0.87,1.50)	1.10 (0.49,2.45)
	IHD	0.99 (0.71,1.38)	1.10 (0.42,2.86)
	HPD	1.56 (0.50,4.83)	0.98 (0.10,5.60)

Notes: Percentiles (1st, 10th, 90th and 99th) in Beijing during cold and warm periods were −7.6 °C, −3.6 °C, 28.1 °C and 30.3 °C, respectively. Percentiles (1st, 10th, 90th and 99th) in Shanghai during cold and warm periods were −0.4 °C, 2.3 °C, 30.3 °C and 33.1 °C, respectively; ^a^ *p* < 0.05.

### 3.5. The Hot and Cold Effects of Temperature for Older People Who Died from Cause-Specific Cardiovascular Diseases

**Figure 3:**
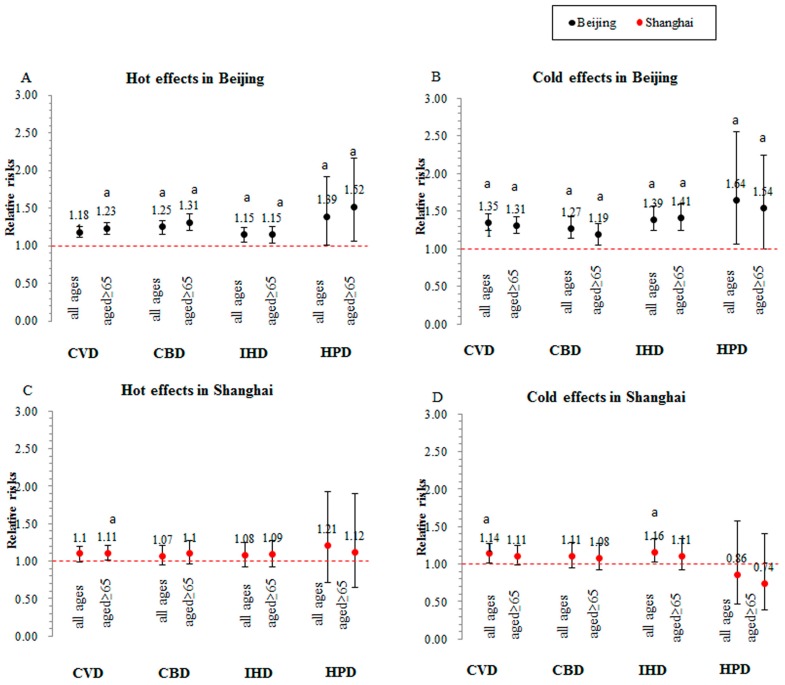
The cumulative hot effects at lag 0–14 days and cold effects at lag 0–27 days on cause-specific cardiovascular mortality among all ages and those aged ≥ 65 years in Beijing and Shanghai, China; ^a^ *p* < 0.05.

Figure 3 shows the hot effects at lag 0–14 days on cause-specific cardiovascular disease mortality for all ages and those aged ≥ 65 years old in the two cities, as well as the cold effects at lag 0–27 days. Compared to the group of all ages, those aged over 65 years old presented higher relative risks for the hot effects of the extremely high temperature in Beijing. The cold effects are similar among all ages and those aged ≥ 65 years in the two cities.

## 4. Sensitivity Analysis

The estimated relationship between temperature and mortality from cardiovascular disease did not change when using different degrees of freedom per year for time (3–6 per year) in Beijing and Shanghai, China (Figure A1). There was also no change when we fitted a time lag of 28–30 days, which also gives similar results (Figure A2).

We also changed the cold and hot thresholds by using the 25th percentile of temperature as the cold thresholds and the 75th percentile of temperature as the hot thresholds, and we estimated the relative risks of extremely low temperature (first percentile of temperature) and extremely high temperature (99th percentile of temperature) on cause-specific cardiovascular mortality, which also were similar to the original estimates (Table A2 and Table A3). We also used the apparent temperature as the meteorological indicator for the two cities to examine the effects of temperature, and we found that the results were similar to the original estimates (Figure A3). The effects of temperature on cause-specific cardiovascular mortality in the two cities after excluding the influences of air pollution in the model were similar to the original results (Figure A4).

## 5. Discussion

This study examined the relationship between the extreme temperatures and the cause-specific cardiovascular mortality in Beijing and Shanghai, China. The associations of temperature with the cause-specific cardiovascular mortality were U-shaped for Beijing and J-shaped for Shanghai, where the optimum temperature was 25 °C and 28 °C, respectively. We observed stronger cold and hot effects in Beijing than in Shanghai. Among the cause-specific cardiovascular mortality in Beijing, we observed the strongest cold and hot effects for HPD mortality; while for Shanghai, people with IHD showed higher susceptibility to the extremely low temperatures than the other types of cardiovascular disease. In general, the low temperature caused greater adverse effects on cause-specific cardiovascular mortality in the two cities.

Many previous studies have reported the U-shaped or J-shaped associations between temperature and mortality [7,8]. Similar results were observed in this study. Beijing presented the “U” shape, with increased relative risks both at low and high temperatures. The relationships between mean temperature and cause-specific cardiovascular mortality in Shanghai were “J” shaped, with increased relative risks at low temperatures. The optimum temperature for cause-specific cardiovascular mortality in Beijing was 25 °C, lower than that in Shanghai (28 °C). Gasparrini *et al*. [29] examined the temperature-mortality associations for 384 locations across the world and found that the minimum mortality temperature was close to the 83th (25.6 °C) percentile of the temperature in Beijing, China, which was consistent with our results. Similarly, Zhang *et al.* [15] reported that the temperature threshold was 26.9 °C in Shanghai, China. This finding is consistent with the optimum temperature in communities with colder climates being lower than in communities with warmer climates [30].

Our results showed that the cold and hot effects of temperatures on cause-specific cardiovascular mortality were stronger in Beijing than those in Shanghai. Similarly, a previous study has observed the stronger cold and hot effects of extreme temperatures on CBD mortality in Beijing than those in Shanghai [14]. Guo *et al*. [12] and Zhang *et al.* [15] also found that the hot effects in Beijing were stronger than those in Shanghai. However, the cold effects in their studies were not consistent with our results, which is likely because of their using the mortality data from one district in each city rather than the population mortality. Therefore, our findings could be explained by two aspects. One possible explanation for this is that the people living in Shanghai with a subtropical climatic pattern are more acclimated to high temperatures. Lee *et al*. [31] found that the tropical natives can maintain cardiac output at lower heart rates with the effects of heat acclimatization. Additionally, the heat acclimatization training can improve the adaptability of the human body to extreme hot environments [32]. However, since Beijing has cooler summers, people living in Beijing are less likely to have or use air conditioners in their homes because of the temperate climate [33]. Therefore, people may be more sensitive to high temperatures; the relative risks increased when exposed to the high temperatures in Beijing. On the other hand, the concentration of ambient PM_10_ in Beijing was higher than that in Shanghai during this study period. It has been reported that air pollutants could trigger acute cardiac events, particularly in people already compromised by CVD [34]. Thus, it is biologically plausible that a high concentration of PM_10_ enhances the adverse effects of extreme temperatures on cardiovascular mortality [35,36]. Besides, air pollution plays a significant independent role in vitamin D deficiency for adults [37]. Epidemiological studies have proven that vitamin D can increase cardiovascular risk [38]. Furthermore, many other socioeconomic factors (e.g., lifestyle, economic status and education levels), disease history and medications may contribute to the differences in the two cities; however, these issues need further investigations.

An important finding of our study is that the susceptibility related to the extreme temperatures varies with the primary causes of death. People with hypertensive disease in Beijing showed the greatest relative risks to both the extremely low and high temperatures. Moreover, the deaths of IHD in Shanghai showed higher susceptibility to the extreme low temperature regarding specific mortality causes. Only a few studies have examined the susceptibility to ambient temperature according to the causes of death outside China. In Korea, the burdens of HPD were greater than the burdens of ischemic heart disease and cerebrovascular disease during heat waves [39]. Several previous studies have proven that blood pressure would increase sharply during the winter season with low temperatures. Recently, Yang *et al.* [40] examined the association between outdoor temperature, blood pressure and CVD mortality among 23,000 individuals with diagnosed CVD, which proposed that mean systolic blood pressure was significantly higher in winter than in summer (145 *vs*. 136 mmHg, *p* < 0.001). They also found above 5 °C, each 10 °C lower outdoor temperature was associated with 6.2 mmHg higher systolic blood pressure. Thus, the awareness of subpopulations that are particularly vulnerable to extreme temperatures is of great concern for public health.

We found that the cold effects during the cold period became weaker compared to the cold effects for the whole year in Beijing. However, the hot effects during the warm period became stronger compared to the hot effects for the whole year in Beijing. Previous studies have shown that both personal level environmental temperature and seasonality are independent predictors of blood pressure (BP) values [41]. Our results were consistent with these findings. Although ambient low temperature increases the daytime BP values [41], there is also evidence that indoor heating might reduce such an impact through raising personal-level environmental temperatures [42], considering that 78% of houses in urban Beijing have central heating [43]. Thus, the cold effects during the cold period in Beijing became weaker. However, the stronger hot effect during the warm season can be explained from three aspects. Firstly, hot days during the warm season are associated with increased nocturnal BP levels [41]. Secondly, the high temperatures during the warm season could decrease the heart rate variability via autonomic nervous system dysfunction [44]. Finally, in this study, we observed the short-term effect of mortality displacement, which was also known as the harvesting effect (Figure A5). After the onset of the extremely high temperatures, the effects on cause-specific cardiovascular mortality were followed by a decrease in mortality below expected levels (around lag 10), which means that the cumulative effects of high temperatures reached the strongest at lag 0–10 days. Thus, the harvesting effect of high temperatures could bring forward deaths among people with cardiac diseases [45]. The present study found a higher susceptibility for the elderly (≥65 years old) to the extremely high temperatures in Beijing. The effects of extremely low temperatures on mortality are similar among all ages and the elderly (≥65 years old). The elderly often suffer from chronic conditions and are usually compromised by extreme temperatures. When the elderly are exposed to heat, they may have physiological changes in thermoregulation and homoeostasis [9,25,46]. However, other factors, such as behavioral patterns in cold weather (*i.e.*, spending less time outdoors or wearing warm clothes), may have protective effects against cold weather [47].

Several studies show that the CVD mortality would increase during both the extremely low and high temperatures [48,49]. In this study, we also found both cold and hot effects on cause-specific cardiovascular mortality in the two cities. The possible biological mechanism underlying the cold and hot effects of temperature on CVD mortality may be explained as follows. It is assumed that cold stress is associated with a rise in blood pressure and an accumulation of thrombogenic factors, such as blood cell counts, plasma cholesterol, C-reactive protein, fibrinogen concentrations and platelet reactivity [50,51]. Besides, exposure to high temperatures may cause vasodilation, improve sweating and increase blood viscosity and cholesterol levels [52].

We used the daily mean temperature as the exposure metric, because it represents the exposure throughout the whole day and night [25]. Previous studies have proven that the mean temperature was either the best predictor of mortality or none of the temperature measures were superior over the others [10]. There were several limitations to our study. First, the data are only collected from two cities in China, so their generalization to China as a whole may be limited. Second, we used the data of temperature and air pollution from sparse stations rather than individual exposures, which might have some measurement error. However, it is likely to be random, not related to the deaths from cardiovascular disease [53]. Third, using the death registry data will likely result in some misclassification of the cause contribution. In this study, we did not adjust the influence of influenza, because there was no pandemic influenza in the two cities during 2007–2009. In particular, since 2007, Beijing has provided free annual influenza vaccination for adults over 60 years old and school-age children. Shanghai also has implemented this plan since 15 October 2009. Moreover, many studies of the effects of temperature on mortality in China [12,13,14,15,35] have not considered the influence of influenza. Even some multi-country studies [29,30] have not considered this influence in their studies. Therefore, we think the model that we used in this study is sufficient to reflect the effect of temperature on cause-specific cardiovascular mortality.

The major strengths of our study include examining the effects of extreme temperatures on CVD, CBD, IHD and HPD simultaneously in cities with different climatic types in China. Our study findings suggest that the people living in a temperate climate area like Beijing may have greater hot effects than the people living in a subtropical area like Shanghai, because they are more acclimated to a higher temperature. The results from the present analyses also suggest that we should pay attention to those people with HPD during the summer with extremely high temperatures. During the winter with extremely low temperatures, we also should pay more attention to those people with HPD in Beijing and people with IHD in Shanghai.

## 6. Conclusions

In this study, both low and high temperatures increased the risk of deaths from CVD, CBD, IHD and HPD in Beijing and Shanghai, China. People in Beijing seem to be more sensitive to the effects of both high and low temperatures than the people in Shanghai. We also identified that those with HPD in Beijing are particularly susceptible to extreme high temperatures. In addition, we should pay more attention to those with IHD in Shanghai during extremely cold days. Low temperature was associated with greater risk of mortality from CVD, CBD, IHD and HPD compared to high temperature.

## Abbreviations

CVD: cardiovascular disease
CBD: cerebrovascular disease
IHD: ischemic heart disease
HPD: hypertensive disease
RH: relative humidity
MT: mean temperature
PM_10_: aerodynamic diameter less than 10 microns
DLNM: distributed lag non-linear model
AIC: Akaike information criterion

## Appendix

**Table A1:** The selected sociodemographic characteristics for Beijing and Shanghai in China (2009).

Factors *	Beijing	Shanghai
Population (million)	17.55	19.21
Birth rate (‰)	8.06	8.64
Mortality rate (‰)	4.56	5.94
Natural growth rate (‰)	3.50	2.70
Age structure (proportion, %)		
0–14	9.90	7.56
15–64	79.98	78.36
≥65	10.09	14.08
Life expectancy	76.10	78.14
Education level (proportion, %)		
unschooled	3.10	4.10
Primary school	13.00	13.30
Junior middle school	30.30	33.80
High school	22.80	25.20
College or above	30.80	23.60

Note: ***** collected from China Statistical Yearbook (2010).

**Table A2:** The cumulative hot effects on mortality from cardiovascular disease, cerebrovascular disease, ischemic heart disease and hypertensive disease in Beijing and Shanghai, China.

City	Lag	Hot Effects *			
		Cardiovascular	Cerebrovascular	Ischemic Heart Disease	Hypertensive Disease
Beijing	0	1.04 (1.02, 1.06) ^a^	1.06 (1.02, 1.09) ^a^	1.04 (1.01, 1.07) ^a^	1.03 (0.93, 1.14)
	0–7	1.26 (1.18, 1.35) ^a^	1.37 (1.25, 1.51) ^a^	1.21 (1.10, 1.34) ^a^	1.32 (0.90, 1.93)
	0–14	1.26 (1.16, 1.37) ^a^	1.38 (1.23, 1.55) ^a^	1.22 (1.07, 1.37) ^a^	1.38 (0.86, 2.19)
	0–27	1.15 (1.03, 1.29) ^a^	1.27 (1.09, 1.50) ^a^	1.13 (0.95, 1.33)	1.32 (0.69, 2.52)
Shanghai	0	1.01 (0.98, 1.04)	0.99 (0.95, 1.03)	1.03 (0.98, 1.09)	1.03 (0.87, 1.23)
	0–7	1.08 (0.98, 1.20)	1.00 (0.87, 1.15)	1.15 (0.98, 1.36)	1.30 (0.74, 2.28)
	0–14	1.10 (0.98, 1.24)	1.05 (0.90, 1.22)	1.12 (0.92, 1.35)	1.22 (0.64, 2.30)
	0–27	1.01 (0.87, 1.19)	1.01 (0.81, 1.24)	0.98 (0.92, 1.35)	1.02 (0.42, 2.46)

Notes: ***** The relative risk of mortality from cardiovascular disease, cerebrovascular disease, ischemic heart disease and hypertensive disease were associated with the 99th percentile of temperature against the 75th percentile of temperature. The 99th percentile of temperature in Beijing and Shanghai was 29.8 °C and 32.4 °C, respectively. The 75th percentile of temperature in Beijing and Shanghai was 24.1 °C and 25.2 °C, respectively; ^a^ *p* < 0.05.

**Table A3:** The cumulative cold effects on cardiovascular disease, cerebrovascular disease, ischemic heart disease and hypertensive disease in Beijing and Shanghai, China.

City	Lag	Cold Effects *			
		Cardiovascular	Cerebrovascular	Ischemic Heart Disease	Hypertensive Disease
Beijing	0	1.02 (1.00, 1.05) ^a^	1.01 (0.98, 1.04)	1.04 (1.00, 1.07) ^a^	1.06 (0.92, 1.21)
	0–7	1.21 (1.11, 1.31) ^a^	1.16 (1.04, 1.30) ^a^	1.23 (1.10, 1.38) ^a^	1.52 (0.97, 2.37)
	0–14	1.38 (1.26, 1.51) ^a^	1.33 (1.17, 1.52) ^a^	1.36 (1.19, 1.55) ^a^	1.68 (1.02, 2.78) ^a^
	0–27	1.60 (1.44, 1.78) ^a^	1.48 (1.28, 1.72) ^a^	1.64 (1.41, 1.92) ^a^	2.53 (1.41, 4.53) ^a^
Shanghai	0	1.01 (0.98, 1.04)	1.00 (0.96, 1.03)	1.03 (0.99, 1.08)	0.99 (0.86, 1.14)
	0–7	1.09 (1.00, 1.21) ^a^	1.07 (0.94, 1.21)	1.16 (0.99, 1.35)	0.93 (0.56, 1.55)
	0–14	1.17 (1.06, 1.30) ^a^	1.20 (1.04, 1.38) ^a^	1.15 (0.97, 1.16)	0.86 (0.49, 1.52)
	0–27	1.31 (1.15, 1.49) ^a^	1.23 (1.03, 1.38) ^a^	1.35 (1.09, 1.66) ^a^	0.91 (0.45, 1.84)

Notes: ***** The relative risk of mortality from cardiovascular disease, cerebrovascular disease, ischemic heart disease and hypertensive disease were associated with the 1st percentile of temperature against the 25th percentile of temperature. The 1st percentile of temperature in Beijing and Shanghai was −6.1 °C and 0.5 °C, respectively. The 25th percentile of temperature in Beijing and Shanghai was 3 °C and 9.8 °C, respectively; ^a^ *p* < 0.05.

## References

[1] Intergovernmental Panel on Climate Change (2013). Climate Change 2013: The Physical Science Basis.

[2] Basagana X., Sartini C., Barrera-Gomez J., Dadvand P., Cunillera J., Ostro B., Sunyer J., Medina-Ramon M. (2011). Heat waves and cause-specific mortality at all ages. Epidemiology.

[3] Turner L.R., Barnett A.G., Connell D., Tong S.L. (2012). Ambient temperature and cardiorespiratory morbidity: A systematic review and meta-analysis. Epidemiology.

[4] Vasconcelos J., Freire E., Almendra R., Silva G.L., Santana P. (2013). The impact of winter cold weather on acute myocardial infarctions in Portugal. Environ. Pollut..

[5] Madrigano J., Mittleman M.A., Baccarelli A., Goldberg R., Melly S., von Klot S., Schwartz J. (2013). Temperature, myocardial infarction, and mortality: Effect modification by individual- and area-level characteristics. Epidemiology.

[6] Hu S.S., Kong L.Z., Gao R.L., Zhu M.L., Wang W., Wang Y.J., Wu Z.S., Chen W.W., Liu M.B. (2012). Outline of the report on cardiovascular disease in China, 2010. Biomed. Environ. Sci..

[7] Curriero F.C., Heiner K.S., Samet J.M., Zeger S.L., Strug L., Patz J.A. (2002). Temperature and mortality in 11 cities of the eastern United States. Am. J. Epidemiol..

[8] McMichael A.J., Wilkinson P., Kovats R.S., Pattenden S., Hajat S., Armstrong B., Vajanapoom N., Niciu E.M., Mahomed H., Kingkeow C. (2008). International study of temperature, heat and urban mortality. the “ISOTHURM” project. Int. J. Epidemiol..

[9] Analitis A., Katsouyanni K., Biggeri A., Baccini M., Forsberg B., Bisanti L., Kirchmayer U., Ballester F., Cadum E., Goodman P.G. (2008). Effects of cold weather on mortality: Results from 15 European cities within the PHEWE project. Am. J. Epidemiol..

[10] Anderson B.G., Bell M.L. (2009). Weather-related mortality: How heat, cold, and heat waves affect mortality in the United States. Epidemiology.

[11] Son J.Y., Lee J.T., Anderson G.B., Bell M.L. (2011). Vulnerability to temperature-related mortality in Seoul, Korea. Environ. Res. Lett..

[12] Guo Y.M., Li S.S., Zhang Y.S., Armstrong B., Jaakkola J.J.K., Tong S.L., Pan X.C. (2013). Extremely cold and hot temperatures increase the risk of ischaemic heart disease mortality: Epidemiological evidence from China. Heart.

[13] Ma W.J., Yang C.X., Chu C., Li T.T., Tan J.G., Kan H.D. (2015). The temperature-mortality relationship in China: An analysis from 66 Chinese communities. Environ. Res..

[14] Chen R., Wang C., Meng X., Chen H., Thach T.Q., Wong C.M., Kan H. (2013). Both low and high temperature may increase the risk of stroke mortality. Neurology.

[15] Zhang Y., Li S., Pan X., Tong S., Jaakkola J.J., Gasparrini A., Guo Y., Wang S. (2014). The effects of ambient temperature on cerebrovascular mortality: An epidemiologic study in four climatic zones in China. Environ. Health.

[16] Guo Y., Barnett A.G., Pan X., Yu W., Tong S. (2011). The impact of temperature on mortality in Tianjin, China: A case-crossover design with a distributed lag nonlinear model. Environ. Health Perspect..

[17] Goggins W.B., Chan E.Y., Yang C., Chong M. (2013). Associations between mortality and meteorological and pollutant variables during the cool season in two Asian cities with sub-tropical climates: Hong Kong and Taipei. Environ. Health.

[18] Davídkovová H., Plavcová E., Kynčl J., Kyselý J. (2014). Impacts of hot and cold spells differ for acute and chronic ischaemic heart diseases. BMC Public Health.

[19] Lin Y.K., Chang C.K., Wang Y.C., Ho T.J. (2013). Acute and prolonged adverse effects of temperature on mortality from cardiovascular diseases. PLoS One.

[20] Medina-Ramon M., Zanobetti A., Cavanagh D.P., Schwartz J. (2006). Extreme temperatures and mortality: Assessing effect modification by personal characteristics and specific cause of death in a multi-city case-only analysis. Environ. Health Perspect..

[21] Grjibovski A.M., Nurgaliyeva N., Kosbayeva A., Menne B. (2012). No association between temperature and deaths from cardiovascular and cerebrovascular diseases during the cold season in Astana, Kazakhstan—The second coldest capital in the world. Int. J. Circumpolar Health.

[22] Zacharias S., Koppe C., Mücke H.G. (2014). Influence of heat waves on ischemic heart diseases in Germany. Climate.

[23] Gasparrini A., Armstrong B., Kenward M.G. (2010). Distributed lag non-linear models. Stat. Med..

[24] Gasparrini A. (2011). Distributed lag linear and non-linear models in R: The package DLNM. J. Stat. Softw..

[25] Breitner S., Wolf K., Peters A., Schneider A. (2014). Short-term effects of air temperature on cause-specific cardiovascular mortality in Bavaria, Germany. Heart.

[26] Armstrong B. (2006). Models for the relationship between ambient temperature and daily mortality. Epidemiology.

[27] Luo Y., Zhang Y.H., Liu T., Rutherford S., Xu Y.J., Xu X.J., Wu W., Xiao J.P., Zeng W.L., Chu C. (2013). Lagged effect of diurnal temperature range on mortality in a subtropical megacity of China. PLoS ONE.

[28] Akaike H. (1987). Factor analysis and AIC. Psychometrika.

[29] Gasparrini A., Guo Y., Hashizume M., Lavigne E., Zanobetti A., Schwartz J., Tobias A., Tong S., Rocklov J., Forsberg B. (2015). Mortality risk attributable to high and low ambient temperature: A multicounty observational study. Lancet.

[30] Guo Y., Gasparrini A., Armstrong B., Li S., Tawatsupa B., Tobias A., Lavigne E., de Sousa Zanotti Stagliorio Coelho M., Leone M., Pan X. (2014). Global variation in the effects of ambient temperature on mortality: A systematic evaluation. Epidemiology.

[31] Lee J.K.W., Nio A.Q.X., Fun D.C.Y., Teo Y.S., Chia E.V., Lim C.L. (2012). Effects of heat acclimatisation on work tolerance and thermoregulation in trained tropical natives. J. Therm. Biol..

[32] Tian Z.X., Li S.S., Zhang J.L., Jaakkola J.J.K., Guo Y.M. (2011). Experimental study on physiological and psychological effects of heat acclimatization in extreme hot environments. Bldg. Environ..

[33] Kovats R.S., Hajat S. (2008). Heat stress and public health: A critical review. Annu. Rev. Public Health.

[34] Gold D.R., Samet J.M. (2013). Air pollution, climate, and heart disease. Circulation.

[35] Li L., Yang J., Guo C., Chen P.Y., Ou C.Q., Guo Y. (2015). Particulate matter modifies the magnitude and time course of the non-linear temperature-mortality association. Environ. Pollut..

[36] Ren C., Williams G.M., Tong S. (2006). Does particulate matter modify the association between temperature and cardiorespiratory disease?. Environ. Health Perspect..

[37] Hosseinpanah F., Pour S.H., Heibatollahi M., Moghbel N., Asefzade S., Azizi F. (2010). The effects of air pollution on vitamin D status in healthy women: A cross sectional study. BMC Public Health.

[38] Lee J.H., O’Keefe J.H., Bell D., Hensrud D.D., Holick M.F. (2008). Vitamin D deficiency an important, common, and easily treatable cardiovascular risk factor?. J. Am. Coll. Cardiol..

[39] Yoon S.J., Oh I.H., Seo H.Y., Kim E.J. (2014). Measuring the burden of disease due to climate change and developing a forecast model in South Korea. Public Health.

[40] Yang L., Li L., Lewington S., Guo Y., Sherliker P., Bian Z., Collins R., Peto R., Liu Y., Yang R. (2015). Outdoor temperature, blood pressure, and cardiovascular disease mortality among 23,000 individuals with diagnosed cardiovascular diseases from China. Eur. Heart J..

[41] Modesti P.A., Morabito M., Massetti L., Rapi S., Orlandini S., Mancia G., Gensini G.F., Parati G. (2013). Seasonal blood pressure changes: An independent relationship with temperature and daylight hours. Hypertension.

[42] Saeki K., Obayashi K., Kurumatani N. (2015). Short-term effects of instruction in home heating on indoor temperature and blood pressure in elderly people: A randomized controlled trial. J. Hypertension.

[43] Beijing Zhongguan Center for Economic Survey Investigation Report for Beijing Residents’ Concerns. http://wwwbeinetnetcn/fxyj/zgdc/201001/t537518html.

[44] Zanobetti A., O’Neill M.S., Gronlund C.J., Schwartz J.D. (2013). Susceptibility to mortality in weather extremes: Effect modification by personal and small-area characteristics. Epidemiology.

[45] Qiao Z., Guo Y., Yu W., Tong S. (2015). Assessment of short- and long-term mortality displacement in heat-related deaths in Brisbane, Australia, 1996–2004. Environ. Health Perspect..

[46] Gasparrini A., Armstrong B., Kovats S., Wilkinson P. (2012). The effect of high temperatures on cause-specific mortality in England and Wales. Occup. Environ. Med..

[47] Yu W., Hu W., Mengersen K., Guo Y., Pan X., Connell D., Tong S. (2011). Time course of temperature effects on cardiovascular mortality in Brisbane, Australia. Heart.

[48] Applegate W.B., Runyan J.W., Brasfield L., Williams M.L., Konigsberg C., Fouche C. (1981). Analysis of the 1980 heat wave in Memphis. J. Am. Geriatr. Soc..

[49] Mercer J.B. (2003). Cold—An underrated risk factor for health. Environ. Res..

[50] Carder M., McNamee R., Beverland I., Elton R., Cohen G.R., Boyd J., Agius R.M. (2005). The lagged effect of cold temperature and wind chill on cardiorespiratory mortality in Scotland. Occup. Environ. Med..

[51] Ballester F., Corella D., Perez-Hoyos S., Saez M., Hervas A. (1997). Mortality as a function of temperature: A study in Valencia, Spain, 1991–1993. Int. J. Epidemiol..

[52] McGeehin M.A., Mirabelli M. (2001). The potential impacts of climate variability and change on temperature-related morbidity and mortality in the United States. Environ. Health. Perspect..

[53] Guo Y., Barnett A.G., Tong S. (2013). Spatiotemporal model or time series model for assessing city-wide temperature effects on mortality?. Environ. Res..

